# Using disease‐selective cell lines to investigate tau strain biology

**DOI:** 10.1002/alz.089601

**Published:** 2025-01-03

**Authors:** Sara A. M. Holec, Christine K. Brown, Matthew P. Frost, Amanda L. Woerman

**Affiliations:** ^1^ Colorado State University, Fort Collins, CO USA; ^2^ University of Massachusetts Amherst, Amherst, MA USA; ^3^ University of Connecticut School of Medicine, Farmington, CT USA

## Abstract

**Background:**

In tauopathies, the protein tau misfolds into a b‐sheet conformation that self‐templates and spreads throughout the brain causing progressive degeneration. Biological and structural data have shown that the shape, or strain, that tau adopts when it misfolds determines which disease a patient will develop. We previously used HEK293T cells expressing TauRD‐YFP to show that tau strain formation is isoform‐specific. However, the field remains unable to differentiate between strains using the same isoforms, which is a critical requirement for the successful development of strain‐specific diagnostics. In this work, we hypothesize that single point mutations can be used to disrupt the misfolded tau conformations in a disease‐specific manner, enabling differentiation between tauopathies.

**Method:**

We built a panel of HEK293T cells expressing several disease‐linked and novel mutations in 4R tau, which we selected based on mutagenesis modeling aimed at selectively inhibiting replication of tau prions isolated from either corticobasal degeneration (CBD) or progressive supranuclear palsy (PSP) patient samples. Infection conditions in the cell lines were optimized for a 384‐well plate using tau prions isolated from Tg(MAPT*P301S^+/+^) and non‐transgenic B6/J mice before testing human patient samples. Cells were imaged using the LionheartFX and infection was determined using custom algorithms developed in Gen5 software.

**Result:**

Samples isolated from control mice do not cause aggregation in the cell lines tested. By comparison, tau prions isolated from Tg(MAPT*P301S^+/+^) mice cause aggregation in some cells, but other point mutations inhibited tau prion replication. Intriguingly, the same mutations inhibited replication of tau prions isolated from PSP patient samples, but not CBD samples. Notably, within this targeted region of the protein, the cryo‐EM structures of tau fibrils in PSP patients and Tg(MAPT*P301S^+/+^) mice show some homology.

**Conclusion:**

We have developed two cell lines that selectively replicate tau prions isolated from CBD patient samples. To our knowledge, this is the first assay capable of differentiating between two different 4R tau prion strains, representing a significant advancement towards the development of disease‐specific diagnostic tools.

